# Enzymatic Treatment of Spent Green Tea Leaves and Their Use in High-Fibre Cookie Production

**DOI:** 10.17113/ftb.60.03.22.7474

**Published:** 2022-09

**Authors:** Ngoc Doan Trang Nguyen, Thi Thuy Huong Phan, Thi Thu Tra Tran, Nu Minh Nguyet Ton, Dinh Le Tam Vo, Van Viet Man Le

**Affiliations:** 1 Department of Food Technology, Ho Chi Minh City University of Technology (HCMUT), 268 Ly Thuong Kiet Street, District 10, Ho Chi Minh City, Vietnam; 2 Vietnam National University, Ho Chi Minh City (VNU-HCM), Linh Trung Ward, Thu Duc City, Ho Chi Minh City, Vietnam

**Keywords:** antioxidant activity, cookie preparation, dietary fibre, enzymatic treatment, spent green tea leaves

## Abstract

**Research background:**

By-products of food industry have been studied as sources of high fibre and antioxidant ingredients for healthy food products, because of their economic and environmental benefits. However, the soluble dietary fibre content of these materials is usually lower than the recommended value that is claimed to bring positive health effects. Enzymatic treatment could be an efficient method for modifying insoluble and soluble dietary fibre contents of these materials. The purpose of this study is to investigate the effects of enzymatic treatment conditions on soluble, insoluble and total dietary fibre mass fractions in spent green tea leaves, and evaluate the quality of dough and cookies when different mass fractions of untreated and treated leaves were added to the recipe.

**Experimental approach:**

The mass fractions of soluble, insoluble and total dietary fibre in spent tea leaf powder was evaluated after the leaves were treated with cellulase amount of 0−25 U/g for 0 to 2 h. Wheat flour was replaced by untreated and treated spent tea leaf powder at 0, 10, 20, 30 and 40% in cookie formulation. Textural properties of dough, proximate composition, physical properties and overall acceptability of cookies were analysed.

**Results and conclusions:**

The appropriate conditions for enzymatic treatment were enzyme loading of 20 U/g and biocatalytic time of 1.5 h, under which the mass fraction of soluble dietary fibre in spent tea leaves increased by 144.5% compared to that of the control sample. The addition of spent tea leaves led to the increase in dough hardness. Increase in the spent tea leaf amount also enhanced fibre mass fraction, antioxidant activity and hardness of cookies but reduced their overall acceptability. Moreover, the enzymatic treatment of spent tea leaves improved the soluble to total dietary fibre ratio of the cookies, which influenced their textural properties and health benefits. The cookies with added 20% untreated or treated spent tea leaves were overall accepted by the panel.

**Novelty and scientific contribution:**

For the first time, spent tea leaves have been treated with enzymes to improve their soluble to total dietary fibre ratio. The treated spent tea leaves are a new promising high-fibre antioxidant ingredient for cookie preparation.

## INTRODUCTION

Cookies are one of the most popular bakery products all around the world thanks to their delicious taste, convenience for the consumption and long shelf life. This product has a high sugar, fat and starch content, providing high energy intake, but low dietary fibre and antioxidant content. Cookies have also been reported to be among the unhealthiest foods that lead to mass gain (*1*). Therefore, the demand for cookies with high fibre content and antioxidant properties is becoming more and more essential. Various ingredients that have a high dietary fibre and antioxidant content have been used in cookie formulation, such as oat flour (*2*), *Murraya koenigii* leaves (*3*) and beetroot leaves (*4*). Recently, by-products from agriculture and food manufacturing industry, such as watermelon rind waste (*5*) and spent coffee grounds (*6*), have also been studied as potential fibre and antioxidant sources for cookie preparation. Among plant by-products, spent green tea leaves have been discovered to be rich in dietary fibre and phenolic compound content, with the total fibre and total phenolic mass fractions of 67.9 and 13.0% (dry mass), respectively (*7*). It has been reported that spent green tea leaves contain gallic acid, epicatechin, epigallocatechin and gallocatechingallate, which have antioxidant properties (*8*). As a vast amount of spent tea leaves is released from the beverage industry every year, this inexpensive and valuable by-product is considered as a promising ingredient for food production.

The potential of utilising spent green tea leaf powder in cookie formulation has recently been recognised. Soma *et al.* (*9*) investigated the impacts of spent green tea powder addition on the quality of biscuits and reported that 7.5% of wheat flour could be replaced by spent tea powder to produce biscuits with enhanced nutritional value and acceptable sensory properties. The mass fractions of soluble (SDF) and insoluble dietary fibre (IDF) in spent tea were 4.07 and 63.69% (dry mass) respectively (*9*), resulting in biscuit products with the soluble, insoluble and total dietary fibre (TDF) mass fractions on dry mass basis of 1.68, 7.73 and 9.41%, respectively, making the ratio of SDF to TDF of nearly 18%. The total polyphenol content as gallic acid equivalents (GAE) of the studied spent tea leaves was 357 mg/100 g and the total content of phenolic acids in the obtained cookies was 29.35 mg/100 g (*9*).

Insoluble and soluble dietary fibre play different roles in human body. While the IDF adsorbs water in the intestine, which helps to increase faecal volume, reduce intestinal transit time and glucose absorption (*10*), the SDF dissolves in water and forms gel, helps to attenuate blood cholesterol and glucose levels, supports digestive health and reduces antibiotic-related problems as a good source of prebiotics (*11*). Besides the TDF content, a well-balanced ratio of SDF to TDF could improve health and well-being. As the SDF to TDF ratio was recommended to be 30-50% (by mass) (*12*), the fibre fractions in spent tea added to cookies studied by Soma *et al*. (*9*) need to be improved in order to enhance their impact on human nutrition. This leads to the necessity of treating spent tea leaves to increase their SDF content before including them into the cookie formulation.

The hydrolysis of IDF in order to enhance the SDF content using enzyme preparations has been reported in the production of different food ingredients from Chinese cabbage waste (*13*) and rice husk (*14*). However, the enzymatic treatment of spent tea leaves to improve their SDF content in order to incorporate them in food products has not been considered. Since cellulose mass fraction makes up the majority of tea fibre composition with 67.7% (lignin and hemicellulose mass fractions are 20.0 and 4.5-7.2%, respectively) (*9*), the appropriate carbohydrase for this ingredient treatment is cellulase. The purpose of our study is to investigate the effects of different enzymatic treatment conditions on the dietary fibre fractions in spent green tea leaves and evaluate the impacts of the untreated and treated leaf powder on the characteristics of dough and cookies.

## MATERIALS AND METHODS

### Materials

Spent green tea leaves were collected after the extraction during green tea beverage production in a factory (Universal Robina Corporation Vietnam, Binh Duong, Vietnam), dried at 60 °C using a convection dryer to reach a moisture mass fraction of 9–11%, ground into powder and sieved through a 40-mesh screen. Commercial wheat flour (Dai Phong Flour Milling Co., Ltd, Can Tho City, Vietnam), eggs (V.Food Ltd, Ho Chi Minh City, Vietnam), unsalted butter (Anchor, Hamilton, New Zealand), table salt, baking powder (Alsa, Schirmeck, France), vanillin powder (Vianco Ltd., Ho Chi Minh City, Vietnam) and isomalt (Beneo, Mannheim, Germany) were procured from the local supermarket. Food grade acesulfame potassium was purchased from Vitasweet Co., Ltd. (Jiangsu, PR China). Celluclast 1.5 L preparation with endoglucanase activity of 80 U/mL (produced by *Trichoderma reesei*) was obtained from Novozymes A/S (Bagsværd, Denmark). One unit of cellulase activity was defined as reducing sugar content that was released from carboxymethylcellulose (CMC) as the substrate of hydrolysis and expressed in U/mL enzyme preparation. The optimal pH and temperature of this enzyme preparation are 4.5–6.0 and 50–60 °C, respectively.

### Enzymatic treatment of spent tea leaves

A mass of 10 g spent tea leaf powder was placed in a 250-mL Erlenmeyer flask. First, the enzyme preparation was mixed with deionised water. The volume of deionised water was calculated so that the moisture content of treated mixture reached, on dry spent tea leaf basis, 7 mL/g. The quantity of enzyme preparation, on dry spent tea leaf basis, was calculated to achieve enzyme loadings of 0, 5, 10, 15, 20 and 25 U/g. The approximate pH value of the treated mixture was 5.0. The Erlenmeyer flask was then covered with aluminium foil and brought into the incubator (model WNB29; Memmert, Schwabach, Germany) for the enzymatic treatment at 50 °C. The treatment time ranged from 0 to 2 h (0, 0.5, 1.0, 1.5 and 2.0 h). At the end of the treatment, the temperature was raised to 95 °C using a water bath and kept constant for 10 min for enzyme inactivation. The enzyme-treated spent tea leaves were dried at 60 °C in the convection dryer to reach a moisture content of 10%, then ground and sieved through a 40-mesh screen. The proximate composition, antioxidant activity, water- and oil-holding capacity of the treated and untreated spent tea leaf powder were measured.

### Cookie manufacturing process

The cookie formula used in this study comprised 100 g flour (wheat flour and spent tea leaf powder), 38.8 g eggs (yolk and white), 58.3 g unsalted butter, 38.8 g isomalt, 0.5 g vanillin powder, 1.3 g baking powder, 0.6 g table salt, 10.8 g water and 0.15 g acesulfame potassium. Ingredients except wheat flour and spent tea leaf powder were mixed together at 200 rpm by a stand mixer (model KitchenAid Artisan 5KSM175PSECA; Whirlpool Corporation, Clyde, OH, USA) for the total of 5 min to obtain a cream mixture. Spent tea leaf powder was blended with mass fractions of wheat flour: 0 (control sample), 10, 20, 30 and 40%. Powder blends were added to the cream mixture and kneaded at 100 rpm for 2 min to prepare the dough. Dough was then split into halves, one half was immediately used for measuring textural properties and the other half was used for making cookies. Dough sheets 4 mm thick were then manually made and cut with a 36-mm diameter round cutter before baking in an electric oven (model VH-259S2D; Sanaky Vietnam Company Limited, Binh Duong, Vietnam). The baking process consisted of two stages, the temperature was set at 175 °C for the first 15 min and lowered to 150 °C for the next 8 min. Baked cookies were then cooled to room temperature in 15 min. After that, the physical and textural properties were evaluated. Cookies were packed in sealed polyethylene pouches and stored for 24 h prior to proximate composition and sensory quality analyses. Polyethylene pouches were sealed with an impulse heat sealer (model M17; Tan Thanh Service Trade Production Co., Ho Chi Minh City, Vietnam).

### Proximate composition

Moisture content was measured using a moisture analyser (model ML-50; A&D Company Limited, Tokyo, Japan). Total lipid content was quantified with Soxhlet extraction using diethyl ether solvent, following AOAC method 930.09 (*15*). Protein content was determined using Kjeldahl method and the protein-nitrogen conversion factor was 5.7 for wheat flour and 6.25 for spent tea leaves and cookies, following AOAC method 979.09 (*16*). Samples were incinerated in an ashing furnace (model AF 11/6B; Lenton Furnaces & Ovens, Hope Valley, UK) at 600 °C to measure ash content according to AOAC method 942.05 (*17*). Starch content was determined using starch digestion method described by Landhäusser *et al*. (*18*) and glucose content was measured using spectrophotometric method with dinitrocyclic acid reagent (*19*). Insoluble (IDF) and soluble dietary fibre (SDF) contents were analysed according to AOAC methods 991.42 (*20*) and 993.19 (*21*), respectively. Total dietary fibre (TDF) content was calculated as the sum of IDF and SDF contents, following AOAC method 991.43 (*22*).

Total phenolics were determined using 1 g sample in 10 mL *φ*(acetone,water)=50% as the solvent for the ultrasound-assisted extraction (VCX 500 Sonicator; Sonics & Materials, Inc., Newtown, CT, USA), following the method described by Bhebhe *et al*. (*23*) and expressed in mg gallic acid equivalents (GAE) per g dry mass. Antioxidant activity was measured with 2,2-diphenyl-1-picryl-hydrazyl-hydrate (DPPH) following the method of Brand-Williams *et al.* (*24*), and Fe(III) reducing antioxidant power (FRAP) following the method of Benzie and Strain (*25*), expressed in mg Trolox equivalents (TE) per g dry mass.

### Water- and oil-holding capacity of materials

Water-holding capacity (WHC) of enzyme-treated spent tea leaf powder, untreated spent tea leaf powder and wheat flour was measured following AACC method 56-30.01 (*26*) with a minor modification. A mass of 3 g sample was mixed with 30 mL water using a vortex for 30 s. After 2 h, the sample tube was centrifuged at 1000×*g* for 20 min. The supernatant was then decanted. The absorbed water was determined by the difference between the sediment and the initial sample mass. Oil-holding capacity (OHC) was measured with the similar process in which samples were mixed with soybean oil (Tuong An Vegetable Oil Joint Stock Company, Ba Ria-Vung Tau Province, Vietnam).

### Texture profile analysis of dough

Texture profile analysis (TPA) of cookie dough was performed using TA.XT*plusC* Texture Analyser (Stable Micro Systems, Godalming, UK). The dough was shaped by a 42 mm diameter and 40 mm height circular mould, compressed up to 25% with the speed of 5 mm/s by a 25 mm diameter cylinder probe. Three recorded textural parameters of dough were hardness (N), springiness and cohesiveness.

### Physical properties of cookies

A set of six cookies was laid edge-to-edge on the platform to measure the average width (*b*/mm). The set was rotated 90° for replicated measurement. A stack of six cookies was measured for the average thickness (*δ*/mm). The spread ratio (*b*/*δ*) was then calculated. The L*, a* and b* values of cookies were measured by Hunter Lab colour Measuring system (CR-400; Konica Minolta, Inc., Tokyo, Japan) and the colour difference against the control sample (*∆E*) was calculated as follows:



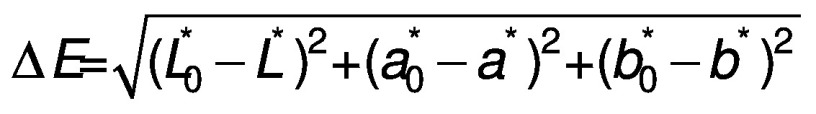



where , and are the colour values of the control, and , and are the colour values of the sample.

The hardness of cookies was evaluated by the breaking strength (N) analysed with three-point break method, using the same instrument that was used to evaluate the TPA of dough and a three-point break rig.

### Overall acceptability of cookies

Overall acceptability of the cookies was evaluated on a 9-point hedonic scale (ranging from 1 for ‘dislike extremely’ to 9 for ‘like extremely’), following the method described by Nguyen *et al*. (*27*). An untrained panel of 60 consumers (34 females and 26 males) aged from 18 to 40 were selected among students and staff in Ho Chi Minh City University of Technology (Ho Chi Minh City, Vietnam). Criteria for recruiting the panellists were that they are regular consumers of cookies and do not have allergies to any food. Samples were served on white plastic plates, coded with different sets of three digits, in random order to all panellists simultaneously.

### Statistical analysis

All experiments were conducted in triplicate and data were reported as mean value±S.D. STATGRAPHICS Centurion 18 software, v. 18.1.12 (*28*) was employed to determine significant differences of mean values (p<0.05), using analysis of variance (ANOVA) with least significant difference (LSD) test. Correlation coefficients r and R^2^ were determined using this software in order to evaluate the significant relationship between variables (p<0.05) when necessary.

## RESULTS AND DISCUSSION

### Effects of enzyme loadings on the fibre fractions of spent tea leaves

**Fig. 1:**
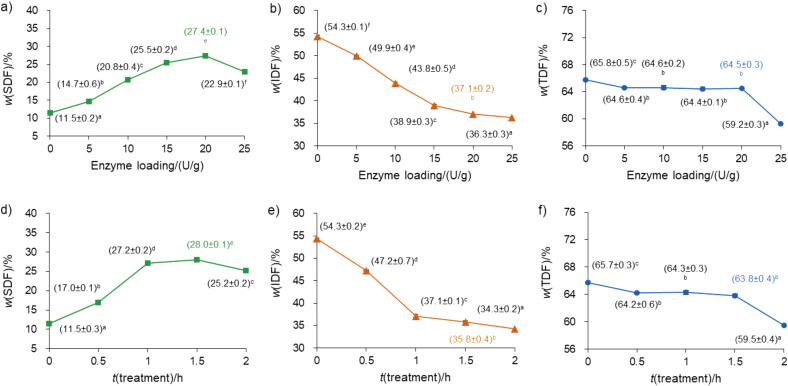
Effect of: a-c) enzyme loading at *γ*(water)=7 mL/g spent tea leaves (dry mass) and *t*=1 h, and d-f) treatment time on mass fractions of soluble dietary fibre (SDF), insoluble dietary fibre (IDF) and total dietary fibre (TDF) in spent tea leaves at *γ*(water)=7 mL/g and enzyme loading 20 U/g. Values followed by different letters for each component differ significantly (p<0.05)

The impact of different enzyme loadings on the soluble (SDF), insoluble (IDF) and total dietary fibre (TDF) contents of spent tea leaves is illustrated in Fig. 1. After 1 h of the treatment, the SDF mass fraction increased by 139.2% (Fig. 1a), while the IDF mass fraction decreased by 31.8% (Fig. 1b) as the enzyme loading rose from 0 to 20 U/g (p<0.05). This was due to the more vigorous cellulolysis of IDF at greater cellulase loadings. However, both SDF and IDF mass fractions decreased as the enzyme loading increased from 20 to 25 U/g. Reduction in SDF mass fraction of spent tea leaves at the high enzyme load could be explained by the degradation of SDF into smaller molecules. Dietary fibre consisting of 3 to 9 monomers is not efficiently precipitated with alcohol, so after filtration the obtained values are low (*29*). It was previously reported that the SDF yield extracted from pineapple pomace increased sharply when the cellulase mass fraction rose from 1 to 5% and decreased moderately when the enzyme content increased to 6% (*30*).

The TDF mass fraction fell by 1.7% when the enzyme load increased from 0 to 5 U/g and remained unchanged as the enzyme load increased from 5 to 20 U/g (Fig. 1c). Nevertheless, further increase in the enzyme load from 20 to 25 U/g reduced the TDF mass fraction by 8.1%. The loss in TDF mass fraction could be due to intensive hydrolysis of SDF into smaller molecules at cellulase loading of 25 U/g.

### Effects of treatment time on the fibre fractions of spent tea leaves

The impact of treatment time on SDF, IDF and TDF mass fractions of spent tea leaves is also shown in Fig. 1. At enzyme loading of 20 U/g, when the treatment time increased from 0 to 1.5 h, the SDF mass fraction rose by 143.5% (Fig. 1d), while the IDF mass fraction fell by 34.0% (Fig. 1e). However, as the biocatalytic time was prolonged from 1.5 to 2.0 h, a decline in the SDF and IDF mass fractions by 9.9 and 4.3%, respectively, was observed. This could be due to the prolonged hydrolysis of SDF to produce low-molecular-mass compounds (*29*). Nguyen *et al*. (*27*) also reported the improvement in the SDF mass fraction of wheat bran when the incubation time rose from 0 to 150 min and its loss when the cellulase treatment lasted from 150 to 210 min.

### Proximate composition, antioxidant and functional properties of the treated and untreated spent tea leaves and wheat flour used in the study

Table 1 shows that the treatment of spent tea leaves with the enzyme loading of 20 U/g for 1.5 h did not change their protein, lipid and ash mass fractions. Both treated and untreated spent tea leaf powders had higher mass fractions of protein, lipid and ash than the wheat flour (p<0.05). The treatment enhanced the SDF mass fraction of the leaves by 145.2%, and decreased the IDF and TDF mass fractions by 34.1 and 3.0%, respectively. Therefore, the SDF to TDF ratio of the treated spent tea leaves was elevated 1.5 times compared to that of the untreated leaves. Decreases in the total phenolic compounds and antioxidant activity of the spent tea leaves were observed due to the oxidation during drying (60 °C). Sagrin and Chong (*31*) reported that drying (40-60 °C) could cause degradation of phenolic compounds in plant leaves. It is clear that both untreated and treated spent tea leaves had significantly higher mass fractions of dietary fibre, phenolic compounds and antioxidant activity than the wheat flour (p<0.05).

**Table 1:** Proximate composition, antioxidant and functional properties of treated, untreated spent tea leaves and wheat flour

Component	Treated spent tea leaves	Untreated spent tea leaves	Wheat flour
*w*(protein)/%	(12.34±0.09)^b^	(12.2±0.1)^b^	(9.64±0.06)^a^
*w*(lipid)/%	(1.74±0.05)^b^	(1.75±0.08)^b^	(1.5±0.1)^a^
w(starch)/%	-	-	(84.8±0.4)
*w*(ash)/%	(3.49±0.03)^b^	(3.47±0.01)^b^	(0.77±0.06)^a^
*w*(TDF)/%	(63.8±0.4)^b^	(65.8±0.7)^c^	(2.76±0.06)^a^
*w*(SDF)/%	(28.00±0.05)^c^	(11.4±0.2)^b^	(1.34±0.04)^a^
*w*(IDF)/%	(35.8±0.4)^b^	(54.4±0.5)^c^	(1.42±0.02)^a^
*ζ*(SDF/TDF)/(g/g)	(43.9±0.3)^b^	(17.4±0.2)^a^	(48.5±0.5)^c^
Total phenolics as *w*(GAE)/(mg/g)*	(84.7±2.8)^b^	(95.9±3.0)^c^	(2.58±0.08)^a^
DPPH scavenging activity as *b*(TE)/(µmol/g)*	(2038±48)^b^	(2488±69)^a^	(10.2±0.5)^c^
Fe(III) reducing power as *b*(TE)/(µmol/g)*	(964±14)^b^	(1108±15)^c^	(3.14±0.08)^a^
WHC/(g/g)*	(4.30±0.04)^b^	(4.5±0.1)^c^	(0.82±0.02)^a^
OHC/(g/g)*	(1.76±0.07)^b^	(2.01±0.03)^c^	(1.18±0.05)^a^

*Results are reported on dry mass basis. Values are presented as mean±standard deviation. Values followed by different letters in the same row differ significantly (p<0.05). TDF=total dietary fibre, SDF=soluble dietary fibre, IDF=insoluble dietary fibre, GAE=gallic acid equivalents, TE=Trolox equivalents, WHC=water-holding capacity, OHC=oil-holding capacity

Moreover, water- and oil-holding capacity of wheat flour were lower than those of untreated and treated spent tea leaves. The enzymatic treatment reduced both WHC and OHC of the leaves by 4.4 and 12.4%, respectively, probably due to the decreased IDF and increased SDF mass fractions. It was also claimed that the cellulase hydrolysis of dietary fibre destroyed the lamellar structure of the IDF, which had higher WHC and OHC than the SDF (*32*). These changes in functional properties of the spent tea leaves after the enzymatic treatment would probably affect dough and cookie quality.

### Textural properties of dough

Hardness, cohesiveness and springiness are among the most important textural properties of solid foods (*33*). The effects of different additions (0, 10, 20, 30 and 40%) of untreated and enzyme-treated spent tea leaves on these properties are shown in Table 2. Overall, increased mass fractions of both untreated and treated spent tea leaf powders added to the recipe significantly enhanced the hardness of cookie dough, but the untreated leaves caused more dramatic increases (p<0.05). As the mass fraction of spent tea leaves reached 40%, the hardness of the dough containing untreated and treated leaves was 3.0 and 2.5 times, respectively, greater than that of the control sample (0%). The hardness of dough relates to its free water content and fibre fractions supplemented from the spent leaves. The incorporation of spent tea leaf powder enhanced the total fibre content and the number of hydroxyl groups in dough system. The interaction between the hydroxyl groups of the fibre and free water molecules *via* hydrogen bonding could cause the increase in the WHC of the dough (*34*, *35*). The reduction of free water amount due to the addition of fibre restricted the mobility of other components in the system and produced a more compact dough (*36*). The enzymatic treatment reduced the IDF content and water-holding capacity of the spent tea leaves. The higher amount of free water molecules in the dough resulted in the lower hardness of the treated spent tea leaves added to the dough.

**Table 2:** Effects of the addition of spent tea leaves on cookie dough properties

Parameter	Control	*x*(untreated tea leaves)/%				*x*(treated tea leaves)/%			
10	20	30	40	10	20	30	40
Hardness/N	(6.0±0.1)^a^	(6.7±0.2)^b^	(7.5±0.1)^c^	(9.5±0.4)^e^	(18.1±0.1)^g^	(6.2±0.2)^a^	(7.2±0.2)^c^	(8.6±0.2)^d^	(15.0±0.3)^f^
Springiness	(0.58±0.01)^f^	(0.47±0.01)^d^	(0.45±0.01)^c^	(0.43±0.01)^bc^	(0.41±0.01)^b^	(0.53±0.03)^e^	(0.45±0.02)^cd^	(0.41±0.02)^ab^	(0.39±0.01)^a^
Cohesiveness	(0.27±0.01)^g^	(0.20±0.01)^d^	(0.18±0.01)^c^	(0.17±0.01)^bc^	(0.14±0.00)^a^	(0.25±0.01)^f^	(0.21±0.02)^e^	(0.18±0.01)^c^	(0.16±0.00)^b^

Values are presented as mean±standard deviation. Values followed by different letters in superscript in the same row differ significantly (p<0.05)

On the other hand, the springiness and cohesiveness had downward trends with the increase of mass fractions of added spent tea leaves. The incorporation of untreated and treated spent tea leaves at 40% caused decreases in the springiness by 29.3 and 32.8%, respectively, compared to the control sample, while the cohesiveness decreased by 48.1 and 40.7%, respectively. The enzymatic treatment of spent tea leaves contributed to a minor increase in the cohesiveness of dough at high tea leaf mass fractions such as 40%. Liu *et al*. (*37*) claimed that the presence of matcha tea fibre inhibited disulfide bonding and the formation of gluten network, leading to the decrease in dough strength. The limitation of gluten network formation and disulfide bond regeneration after being broken by external forces could be an explanation for the decreased cohesiveness of dough and the ability to recover its initial form (springiness). Nevertheless, it was also reported that tea polyphenols caused the breakage of hydrogen bonds, increased the cross-linking of gluten network and interactively arranged gluten chains to build a ’grid’ structure, consequently enhancing the cohesiveness of dough (*37*). Different results were observed probably because of various conditions of dough kneading, including moisture content, kneading temperature and mixing rate. Combined effects of spent tea leaf fibre and polyphenols on the formation of gluten network of cookie dough should be further investigated to clarify their impacts on dough textural properties.

Similar increase in hardness and reduction in springiness and cohesiveness of cookie dough were previously reported when the mass fraction of dried moringa (*Moringa oleifera* Lam.) leaf powder was enhanced from 0 to 15% (*38*). Armero and Collar (*39*) also claimed that dough cohesiveness was negatively correlated with its hardness and positively correlated with its springiness.

### Chemical analysis of cookies

The proximate composition of cookies with spent tea leaves is shown in Table 3. The protein and ash mass fractions of cookies slightly increased, while the starch mass fraction considerably decreased as higher mass fractions of spent tea leaf powder were added (p<0.05). This could be due to the differences between the proximate compositions of spent tea leaf powder and wheat flour. Significant differences were not observed in protein, ash and starch mass fractions between the cookies with added untreated or treated spent tea leaves at similar mass fractions (p>0.05). There were also substantial gains in TDF, SDF and IDF mass fractions of cookies as the mass fraction of added tea leaves increased. The enzymatic treatment of the leaves resulted in higher SDF, lower TDF and IDF mass fractions of cookies. In the cookies with added 30% of untreated spent tea leaves, the TDF, SDF and IDF contents were 8.1, 3.3 and 12.5 times, respectively, higher than the control, while in the cookies with the treated spent tea leaves these values were 7.4, 6.3 and 8.3 times, respectively, higher (p<0.05). The SDF to TDF ratio of cookies with untreated spent tea leaves did not reach the recommended value range (30-50% (*12*)). Interestingly, the cellulase treatment of the leaves successfully improved the SDF to TDF ratio of the cookies with 10 to 40% treated spent tea leaves, to accomplish the recommended ratio for health benefits. As the mass fraction of spent tea leaves increased from 10 to 40%, the SDF to TDF ratio of cookies containing treated tea leaves was enhanced from 1.90 to 2.14 times compared to that of the cookies with the untreated spent tea leaves. In the future, *in vivo* tests should be carried out to confirm healthy impacts of the cookies with both untreated and treated spent tea leaves. However, the cookies with the treated leaves had lower total phenolic content and antioxidant activity than the cookies with the untreated leaves. This could be explained by the degradation of antioxidant compounds in the spent tea leaves during drying after the enzymatic treatment.

**Table 3:** Effects of the addition of spent tea leaves on proximate composition, total phenolic content and antioxidant activity of cookies

Component	Control	*w*(untreated tea leaves)/%				*w*(treated tea leaves)/%			
10	20	30	40	10	20	30	40
*w*(protein)/%*	(8.2±0.2)^a^	(8.7±0.2)^b^	(9.0±0.1)^bc^	(9.5±0.3)^de^	(9.9±0.2)^f^	(8.7±0.3)^b^	(9.2±0.2)^cd^	(9.6±0.2)^ef^	(9.9±0.2)^f^
*w*(lipid)/%*	(24.1±0.3)^a^	(24.33±0.08)^ab^	(24.6±0.1)^bc^	(24.85±0.07)^cd^	(25.1±0.1)^de^	(24.52±0.09)^abc^	(24.8±0.5)^cd^	(25.2±0.5)^de^	(25.4±0.3)^e^
*w*(starch)/%*	(55.6±0.3)^e^	(49.9±0.2)^d^	(45.1±0.2)^c^	(39.2±0.2)^b^	(32.8±0.2)^a^	(49.4±0.2)^d^	(45.3±0.6)^c^	(39.1±0.5)^b^	(32.9±0.5)^a^
*w*(ash)/%*	(1.25±0.03)^a^	(1.35±0.03)^b^	(1.49±0.06)^c^	(1.63±0.05)^d^	(1.72±0.03)^ef^	(1.38±0.02)^b^	(1.52±0.02)^c^	(1.67±0.03)^de^	(1.75±0.05)^f^
*w*(TDF)/%*	(1.42±0.04)^a^	(6.09±0.09)^c^	(8.3±0.2)^e^	(11.51±0.03)^g^	(14.71±0.08)^i^	(4.75±0.08)^b^	(7.5±0.2)^d^	(10.5±0.1)^f^	(14.3±0.3)^h^
*w*(SDF)/%*	(0.68±0.03)^a^	(1.36±0.04)^b^	(1.72±0.03)^c^	(2.25±0.06)^e^	(2.67±0.03)^f^	(2.01±0.08)^d^	(3.18±0.05)^g^	(4.31±0.07)^h^	(5.6±0.1)^i^
*w*(IDF)/%*	(0.74±0.02)^a^	(4.73±0.09)^d^	(6.5±0.1)^f^	(9.25±0.07)^h^	(12.04±0.06)^i^	(2.74±0.05)^b^	(4.3±0.2)^c^	(6.2±0.1)^e^	(8.8±0.2)^g^
*ζ*(SDF/TDF)/(g/g)	(47.63±0.5)^h^	(22.32±0.60)^d^	(20.8±0.2)^c^	(19.6±0.6)^b^	(18.1±0.2)^a^	(42.3±1.2)^g^	(42.5±0.9)^g^	(41.2±0.8)^f^	(38.8±0.1)^e^
TPC as *w*(GAE)/(mg/g)*	(2.63±0.08)^a^	(6.04±0.06)^b^	(13.0±0.2)^d^	(22.2±0.5)^f^	(25.9±0.4)^g^	(5.58±0.06)^b^	(7.9±0.2)^c^	(13.1±0.4)^d^	(15.1±0.2)^e^
DPPH scavenging as *m*(TE)/ (µmol/g)*	(2.14±0.03)^a^	(46.9±1.0)^b^	(75.5±0.9)^e^	(85.9±0.5)^f^	(87.6±0.2)^f^	(47.3±0.6)^b^	(58.6±1.1)^c^	(62.9±1.9)^d^	(72.6±2.3)^e^
Fe(III) reducing power as *m*(TE)/(µmol/g)*	(14.44±0.4)^a^	(76.3±1.6)^d^	(129.3±1.4)^g^	(215.4±1.2)^h^	(246.9±2.3)^i^	(30.2±0.6)^b^	(68.1±2.9)^c^	(94.6±1.0)^e^	(119.4±1.2)^f^

*Results are reported on dry mass basis. Values are presented as mean±standard deviation. Values followed by different letters in superscript in the same row differ significantly (p<0.05). TDF=total dietary fibre, SDF=soluble dietary fibre, IDF=insoluble dietary fibre, TPC=total phenolic compounds, GAE=gallic acid equivalents, TE=Trolox equivalents

### Physical characteristics of cookies

Table 4 shows physical properties including width, thickness, spread ratio, hardness and instrumental colour of the cookies with different types of spent tea leaves. The thickness of cookies slightly grew as the spent tea leaf mass fraction increased, while the width was slightly reduced, resulting in the decreased spread ratio (*b*/*δ*). The spread of cookie during baking is caused by the gravitational flow and the expansion of dough with leavening (*40*). As the dough flows during baking, protein undergoes the ’apparent’ glass transition which allows it to swell and form a network (*41*). The mobility of free water molecules decreases and the dough viscosity increases to a point when it is sufficient to stop the spreading of the dough (*42*). It is suggested that the addition of fibre into the cookie dough lessens the amount of free water to dissolve soluble components, increasing dough viscosity and reducing the spread of the product. Similar results were reported by Younis *et al*. (*43*), when the mass fraction of mosambi (*Citrus limetta*) peel powder added to cookie formula increased from 4 to 12%, the thickness of cookies rose from 9.82 to 11.36 mm while their diameter decreased from 59.10 to 55.12 mm, resulting in the decreased spread ratio from 5.62 to 4.86. It can be noted that the cellulolytic treatment of spent tea leaves did not affect spread ratio of the cookies.

**Table 4:** Effects of the addition of spent tea leaves on physical properties and sensory acceptability of cookies

Parameter	Control	*w*(untreated tea leaves)/%				*w*(treated tea leaves)/%			
10	20	30	40	10	20	30	40
*b*/mm	(35.1±0.3)^c^	(35.2±0.4)^c^	(34.5±0.3)^ab^	(34.3±0.3)^ab^	(34.1±0.2)^a^	(35.3±0.4)^c^	(35.1±0.4)^c^	(35.0±0.4)^c^	(34.5±0.3)^b^
*δ/*mm	(5.75±0.06)^a^	(5.82±0.09)^a^	(6.1±0.1)^c^	(6.25±0.05)^d^	(6.38±0.02)^e^	(5.92±0.08)^b^	(6.1±0.1)^c^	(6.28±0.08)^d^	(6.44±0.05)^e^
*b/δ*	(6.10±0.05)^f^	(6.0±0.1)^ef^	(5.66±0.08)^cd^	(5.49±0.06)^b^	(5.34±0.04)^a^	(6.0±0.1)^e^	(5.7±0.1)^d^	(5.56±0.09)^bc^	(5.36±0.04)^a^
Hardness/N	(9.8±0.5)^a^	(12.0±1.7)^bc^	(14.4±0.4)^d^	(17.3±0.7)^e^	20.9±1.2)^g^	(10.9±0.9)^ab^	(12.6±1.1)^c^	(15.5±1.8)^d^	(18.9±2.0)^f^
L*	(70.67±0.03)^f^	(48.6±0.1)^e^	(44.5±0.4)^d^	(43.5±0.09)^c^	(43.5±0.2)^c^	(48.7±0.4)^e^	(41.2±0.2)^b^	(40.75±0.05)^a^	(40.6±0.2)^a^
a*	(-0.42±0.01)^g^	(-0.92±0.03)^e^	(-1.37±0.02)^c^	(-1.43±0.06)^bc^	(-1.75±0.04)^a^	(-0.83±0.02)^f^	(-0.93±0.02)^e^	(-1.07±0.04)^d^	(-1.47±0.07)^b^
b*	(35.7±0.3)^f^	(19.5±0.4)^e^	(14.9±0.2)^d^	(14.0±0.1)^c^	(13.51±0.09)^b^	(19.7±0.2)^e^	(14.3±0.2)^c^	(13.3±0.4)^b^	(12.9±0.1)^a^
*ΔE*	(0.00±0.00)^a^	(27.4±0.3)^b^	(33.5±0.3)^c^	(34.84±0.04)^d^	(35.2±0.3)^d^	(27.2±0.4)^b^	(36.6±0.4)^e^	(37.4±0.4)^f^	(37.81±0.06)^f^
Acceptability	(6.6±1.4)^e^	(5.9±1.3)^d^	(5.7±1.4)^cd^	(4.3±1.4)^b^	(3.3±1.5)^a^	(6.1±1.5)^d^	(5.4±1.6)^c^	(4.5±1.5)^b^	(3.7±1.6)^a^

Values are presented as mean±standard deviation. Values followed by different letters in the same row differ significantly (p<0.05). *b*=width, *δ*=thickness, *b/δ*=spread ratio, L*=brightness, a*=red/green, b*=yellow/blue, Δ*E*=colour difference against the control sample

As higher mass fractions of wheat flour were replaced by spent tea leaf powder, an upward trend in the hardness of cookies was observed, which was positively correlated with dough hardness (the correlation coefficient r and coefficient of determination R^2^ were 0.89 and 0.80, respectively, for the dough and cookies containing untreated spent tea leaves, and 0.92 and 0.85, respectively, for the dough and cookies with the treated spent tea leaves). Ajila *et al*. (*44*) investigated the impact of mango peel powder on cookie characteristics and reported that the hardness of cookies increased from 8.6 to 19.3 N as the mango peel powder mass fraction rose from 0 to 20%. Another point worth mentioning is that the hardness of cookies with treated spent tea leaves had less considerable difference from the control sample than the hardness of cookies with untreated spent tea leaves. This could be explained by the higher SDF mass fraction of cookies enriched with treated leaves than of those enriched with untreated leaves. Hydrolysed fibre had lower WHC and more free water was available in the dough for the swelling of starch granules, in which the breakage of intramolecular hydrogen bonds and the mobility of macromolecules in dough system were increased, resulting in cookies with lower breaking forces (*36*). Our study confirmed that cookies produced from the dough with greater hardness and cohesiveness tended to have harder texture and lower spreading (*45*).

The addition of 10 to 30% spent tea leaf powder resulted in the darker colour (decreased L* value) of cookies, probably because of dark coloured compounds such as thearubigins, theaflavins and theabrownines, which were formed by the oxidation of spent tea leaf catechins during baking (*46*). The greenness of cookies was enhanced (lower negative value of a*) and the yellowness was reduced (decreased b* value) as higher mass fractions of spent tea leaves were incorporated. This could be explained by the increase in chlorophyll pigment content as tea leaves were added to the formulation. As a result, the colour differences between the cookies with spent tea leaves and the control sample (*∆E*) were enhanced when the leaf mass fraction rose from 10 to 30%. However, *∆E* values of the cookies with 30 and 40% spent tea leaves differed insignificantly (p>0.05). Ahmad *et al*. (*47*) observed decreased lightness of the cookie samples with 4% green tea powder. Moreover, the enzymatic treatment of spent tea leaves also reduced brightness (L*) of cookies, resulting in the greater colour difference against the control sample than the cookies with untreated tea leaves. This might be due to the degradation of chlorophyll into colourless compounds (*48*) and the oxidation of phenolic compounds (*49*) of the enzyme-treated spent tea leaves during drying.

### Overall acceptability of cookies with spent tea leaves

The overall acceptability of cookies with different spent tea leaf mass fractions is shown in Table 4. The overall score fell with the rise in the tea leaf mass fraction and the enzymatic treatment of the leaves did not significantly affect the acceptability of cookies (p>0.05). The control cookies received the highest score (6.6±1.4) and the least accepted samples were cookies with added 40% untreated and treated spent tea leaves (3.3±1.5 and 3.7±1.6, respectively). The highest mass fraction of both types of spent tea leaves accepted by the panellists (score ≥5.0) was 20%. The reason for this downward trend in the acceptability could be the increase in cookie astringency and hardness contributed by the tea leaves. Astringency is considered as a negative contributor to the acceptability of various food products and a major reason for the rejection of plant products by consumers, despite the health benefits from phytonutrients (*50*). Despite the textural improvement of cookies as spent tea leaves were treated with cellulase, significant difference between the overall acceptance of the cookies with similar mass fractions of untreated and treated tea leaves was not observed. The responses to cookie textural preference were reported to be nonpersistent; there were consumers who preferred hard crunchy cookies, while others preferred soft chewy cookies since they were less dry and fresher than the hard ones (*51*). Therefore, the increased hardness of cookies could be an unfavourable attribute for panellists who prefer soft cookies, but a positive characteristic for people who prefer hard cookies.

## CONCLUSIONS

The treatment of spent tea leaves using commercial cellulase preparation with enzyme load of 20 U/g for 1.5 h successfully improved the soluble to total dietary fibre ratio of cookies incorporated with the treated spent tea leaf powder. The soluble to total dietary fibre ratio of the cookies made with untreated spent tea leaves did not reach the recommended value to efficiently provide health benefits. The enzymatic treatment of spent tea leaves elevated the soluble to total dietary fibre ratio of the cookies by 1.90, 2.04, 2.01 and 2.14 times compared to the ones with the untreated leaves, as the spent tea leaf mass fraction rose from 10 to 20, 30 and 40%, respectively, achieving the recommended value of 30-50%. Despite some negative effects of spent tea leaf powder on the quality of cookies such as increased hardness, limited spreading and darker colour, their nutritional value was enhanced with the supplementation of dietary fibre and antioxidant compounds. The cellulase treatment of spent tea leaves decreased the differences in physical properties of the dough and cookies prepared with 100% wheat flour and the cookies with added spent tea leaves. The highest mass fraction of untreated and treated spent tea leaf powder in the cookie formulation that achieved the overall sensory acceptance was up to 20%. Thus, enzymatic treatment was an efficient method for modifying dietary fibre composition of spent green tea leaves. Enzymatically treated spent green tea leaves could be considered as a promising source of dietary fibre and antioxidants that can be utilised in manufacturing value-added food products.

## References

[1] Carels RA Harper J Konrad K. Qualitative perceptions and caloric estimations of healthy and unhealthy foods by behavioral weight loss participants. Appetite. 2006;46(2):199–206. 10.1016/j.appet.2005.12.002 16466830

[2] Lee NY Kang CS. Quality improvement and antioxidant activity of sugar-snap cookies prepared using blends of cereal flour. Prev Nutr Food Sci. 2018;23(2):160–5. 10.3746/pnf.2018.23.2.160 30018895 PMC6047875

[3] Drisya CR Swetha BG Velu V Indrani D Singh RP. Effect of dried *Murraya koenigii* leaves on nutritional, textural and organoleptic characteristics of cookies. J Food Sci Technol. 2013;52(1):500–6. 10.1007/s13197-013-1002-2

[4] Asadi SZ Khan MA. The effect of beetroot (*Beta vulgaris* L.) leaves powder on nutritional, textural, sensorial and antioxidant properties of cookies. J Culin Sci Technol. 2021;19(5):424–38. 10.1080/15428052.2020.1787285

[5] Naknaen P Itthisoponkul T Sondee A Angsombat N. Utilization of watermelon rind waste as a potential source of dietary fiber to improve health promoting properties and reduce glycemic index for cookie making. Food Sci Biotechnol. 2016;25(2):415–24. 10.1007/s10068-016-0057-z 30263285 PMC6049186

[6] Aguilar-Raymundo VG Sánchez-Páez R Gutiérrez-Salomón AL Barajas-Ramírez JA. Spent coffee grounds cookies: Sensory and texture characteristics, proximate composition, antioxidant activity, and total phenolic content. J Food Process Preserv. 2019;43(12):e14223. 10.1111/jfpp.14223

[7] Ramdani D Chaudhry AS Seal CJ. Chemical composition, plant secondary metabolites, and minerals of green and black teas and the effect of different tea-to-water ratios during their extraction on the composition of their spent leaves as potential additives of ruminants. J Agric Food Chem. 2013;61(20):4961–7. 10.1021/jf4002439 23621359

[8] Nadiah NI Uthumporn U. Determination of phenolic and antioxidant properties in tea and spent tea under various extraction method and determination of catechins, caffeine and gallic acid by HPLC. Int J Adv Sci Eng Inf Technol. 2015;5(3):158–64. 10.18517/ijaseit.5.3.520

[9] Soma G Mahadevamma S Sudha ML. Characterisation of tea fiber and its utilisation as a functional ingredient in the preparation of biscuits. Int Food Res J. 2016;23(6):2525–33.

[10] Slavin JL Savarino V Paredes-Diaz A Fotopoulos G. A review of the role of soluble fiber in health with specific reference to wheat dextrin. J Int Med Res. 2009;37(1):1–17. 10.1177/147323000903700101 19215668

[11] Chawla R Patil GR. Soluble dietary fiber. Compr Rev Food Sci. 2010;9(2):178–96. 10.1111/j.1541-4337.2009.00099.x

[12] Schneeman BO. Soluble vs insoluble: Different physiological responses. Food Technol. 1987;41(2):81–2.

[13] Park SY Yoon KY. Enzymatic production of soluble dietary fiber from the cellulose of Chinese cabbage waste and potential use as a functional food source. Food Sci Biotechnol. 2015;24(2):529–35. 10.1007/s10068-015-0069-0

[14] Vegas R Alonso JL Domínguez H Parajó JC. Enzymatic processing of rice husk autohydrolysis products for obtaining low molecular weight oligosaccharides. Food Biotechnol. 2008;22(1):31–46. 10.1080/08905430701863811

[15] Official Method AOAC. 930.09. Ether extract of plants. Gravimetric method. Rockville, MD, USA: AOAC International; 1997.

[16] Official Method AOAC. 979.09. Protein in grains. Kjeldahl method. Rockville, MD, USA: AOAC International; 2000.

[17] Official Method AOAC. 942.05. Ash of animal feed. Rockville, MD, USA: AOAC International; 2005.

[18] Landhäusser SM Chow PS Dickman LT Furze ME Kuhlman I Schmid S Standardized protocols and procedures can precisely and accurately quantify non-structural carbohydrates. Tree Physiol. 2018;38(12):1764–78. 10.1093/treephys/tpy118 30376128 PMC6301340

[19] Sadasivam S, Manickam A. Carbohydrates. In: Sadasivam S, Manickam A. Biochemical methods. New Delhi, India: New Age International Publishers; 2005. pp. 1-21.

[20] Official Method AOAC. 991.42. Insoluble dietary fiber in foods and food products. Enzymatic-gravimetric method. Rockville, MD, USA: AOAC International; 1994.

[21] Official Method AOAC. 993.19. Soluble dietary fiber in foods and food products. Enzymatic-gravimetric method (phosphate buffer). Rockville, MD, USA: AOAC International; 1996.

[22] Official Method AOAC. 991.43. Total, soluble, and insoluble dietary fiber in foods. Enzymatic-gravimetric method. Rockville, MD, USA: AOAC International; 1994.

[23] Bhebhe M Füller TN Chipurura B Muchuweti M. Effect of solvent type on total phenolic content and free radical scavenging activity of black tea and herbal infusions. Food Anal Methods. 2016;9(4):1060–7. 10.1007/s12161-015-0270-z

[24] Brand-Williams W Cuvelier ME Berset C. Use of a free radical method to evaluate antioxidant activity. Lebensm Wiss Technol. 1995;28(1):25–30. 10.1016/S0023-6438(95)80008-5

[25] Benzie IFF, Strain JJ. Ferric reducing/antioxidant power assay: Direct measure of total antioxidant activity of biological fluids and modified version for simultaneous measurement of total antioxidant power and ascorbic acid concentration. In: Packer L, editor. Methods in enzymology. San Diego, CA, USA: Academic Press; 1999. pp. 15-27. 10.1016/S0076-6879(99)99005-5 10.1016/S0076-6879(99)99005-5 9916193

[26] AACC method 56-30.01. Water hydration capacity of protein materials. St. Paul, MN, USA: Cereals & Grains Association; 2000.

[27] Nguyen SN Vien MD Le TTT Tran TTT Ton NMN Le VVM. Effects of enzymatic treatment conditions on dietary fibre content of wheat bran and use of cellulase-treated bran in cookie. Food Sci Technol. 2021;56(8):4017–25. 10.1111/ijfs.15022

[28] Centurion STATGRAPHICS. 18, v. 18.1.12, Statgraphics Technologies Inc, The Plains, VA, USA; 2017. Available from: https://www.statgraphics.com/download18.

[29] Dai FJ Chau CF. Classification and regulatory perspectives of dietary fiber. J Food Drug Anal. 2017;25(1):37–42. 10.1016/j.jfda.2016.09.006 28911542 PMC9333437

[30] Hu H Zhao Q. Optimization extraction and functional properties of soluble dietary fiber from pineapple pomace obtained by shear homogenization-assisted extraction. RSC Advances. 2018;8(72):41117–30. 10.1039/C8RA06928J 35559297 PMC9092029

[31] Sagrin MS Chong G. Effects of drying temperature on the chemical and physical properties of *Musa acuminata* Colla (AAA Group) leaves. Ind Crops Prod. 2013;45:430–4. 10.1016/j.indcrop.2012.12.036

[32] Wen Y Niu M Zhang B Zhao S Xiong S. Structural characteristics and functional properties of rice bran dietary fiber modified by enzymatic and enzyme-micronization treatments. Lebensm Wiss Technol. 2017;75:344–51. 10.1016/j.lwt.2016.09.012

[33] Di Monaco R Cavella S Masi P. Predicting sensory cohesiveness, hardness and springiness of solid foods from instrumental measurements. J Texture Stud. 2008;39(2):129–49. 10.1111/j.1745-4603.2008.00134.x

[34] Chaplin MF. Fibre and water binding. Proc Nutr Soc. 2003;62(1):223–7. 10.1079/PNS2002203 12756971

[35] Rosell CM Rojas JA De Barber CB. Influence of hydrocolloids on dough rheology and bread quality. Food Hydrocoll. 2001;15(1):75–81. 10.1016/S0268-005X(00)00054-0

[36] Šarić B Dapčević-Hadnađev T Hadnađev M Sakač M Mandić A Mišan A Fiber concentrates from raspberry and blueberry pomace in gluten-free cookie formulation: Effect on dough rheology and cookie baking properties. J Texture Stud. 2019;50(2):124–30. 10.1111/jtxs.12374 30345519

[37] Liu Z Chen J Zheng B Lu Q Chen L. Effects of matcha and its active components on the structure and rheological properties of gluten. Lebensm Wiss Technol. 2020;124:109197. 10.1016/j.lwt.2020.109197

[38] Dachana K Rajiv J Indrani D Prakash J. Effect of dried moringa (*Moringa oleifera* Lam) leaves on rheological, microstructural, nutritional, textural and organoleptic characteristics of cookies. J Food Qual. 2010;33(5):660–77. 10.1111/j.1745-4557.2010.00346.x

[39] Armero E Collar C. Texture properties of formulated wheat doughs. Relationships with dough and bread technological quality. Z Lebensm Unters Forsch. 1997;204(2):136–45. 10.1007/s002170050050

[40] Hoseney R, Rogers D. Mechanism of sugar functionality in cookies. In: Faridi H, editor. The science of cookie and cracker production. New York, USA: Chapman & Hall; 1995. pp. 203-25.

[41] Doescher LC Hoseney RC. Effect of sugar type and flour moisture on surface cracking of sugar-snap cookies. Cereal Chem. 1985;62(4):263–6.

[42] Beleia A Miller RA Hoseney RC. Starch gelatinization in sugar solutions. Stärke. 1996;48(7-8):259–62. 10.1002/star.19960480705

[43] Younis K Islam R Jahan K Kundu M Ray A. Investigating the effect of mosambi (*Citrus limetta*) peel powder on physicochemical and sensory properties of cookies. Qual Assur Saf. 2016;8(3):393–8. 10.3920/QAS2015.0706

[44] Ajila CM Leelavathi K Prasada Rao UJS. Improvement of dietary fiber content and antioxidant properties in soft dough biscuits with the incorporation of mango peel powder. J Cereal Sci. 2008;48(2):319–26. 10.1016/j.jcs.2007.10.001

[45] Gujral HS Mehta S Samra IS Goyal P. Effect of wheat bran, coarse wheat flour, and rice flour on the instrumental texture of cookies. Int J Food Prop. 2003;6(2):329–40. 10.1081/JFP-120017816

[46] Ning J Hou GG Sun J Wan X Dubat A. Effect of green tea powder on the quality attributes and antioxidant activity of whole-wheat flour pan bread. Lebensm Wiss Technol. 2017;79:342–8. 10.1016/j.lwt.2017.01.052

[47] Ahmad M Baba WN Wani TA Gani A Gani A Shah U Effect of green tea powder on thermal, rheological & functional properties of wheat flour and physical, nutraceutical & sensory analysis of cookies. J Food Sci Technol. 2015;52(9):5799–807. 10.1007/s13197-014-1701-3 26344994 PMC4554617

[48] Solovchenko A, Yahia EM, Chen C. Pigments. In: Yahia EM, Carrillo-Lopez A, editors. Postharvest physiology and biochemistry of fruits and vegetables. Oxfordshire, UK: Woodhead Publishing; 2018. pp. 225-52. 10.1016/B978-0-12-813278-4.00011-7 10.1016/B978-0-12-813278-4.00011-7

[49] Yu K Zhou HM Zhu KX Guo XN Peng W. Physicochemical changes in the discoloration of dried green tea noodles caused by polyphenol oxidase from wheat flour. Lebensm Wiss Technol. 2020;130:109614. 10.1016/j.lwt.2020.109614

[50] Lesschaeve I Noble AC. Polyphenols: Factors influencing their sensory properties and their effects on food and beverage preferences. Am J Clin Nutr. 2005;81(1):330S–5S. 10.1093/ajcn/81.1.330S 15640499

[51] Jeltema M Beckley J Vahalik J. Model for understanding consumer textural food choice. Food Sci Nutr. 2015;3(3):202–12. 10.1002/fsn3.205 25987995 PMC4431788

